# Complexing Aβ Prevents the Cellular Anomalies Induced by the Peptide Alone

**DOI:** 10.1007/s12031-014-0233-7

**Published:** 2014-03-07

**Authors:** A. G. Henriques, J. M. Oliveira, B. Gomes, R. Ruivo, E. F. da Cruz e Silva, O. A. B. da Cruz e Silva

**Affiliations:** Laboratório de Neurociências, Centro de Biologia Celular, SACS, Universidade de Aveiro, Aveiro, Portugal

**Keywords:** Laminin, Gelsolin, F-actin, Alzheimer’s disease therapeutics, Fibril inhibition, Aggregation

## Abstract

Retention of intracellular secreted APP (isAPP) can be provoked by the neurotoxic peptide Aβ. The latter decreases in the cerebrospinal fluid of Alzheimer’s disease (AD) patients, as a consequence of its cerebral accumulation and deposition into senile plaques. Of similar relevance, secreted APP (sAPP) levels can be associated with AD. The studies here presented, reinforce the link between sAPP and Aβ and address putative therapeutic strategies. Laminin and gelsolin are potential candidates; both prevent Aβ fibril formation by complexing with Aβ, thus attenuating its neurotoxicity. We show that preincubation of Aβ with laminin and gelsolin has the effect of rendering it less potent to isAPP accumulation in cortical neurons. This appears to be related to a decrease in F-actin polymerization, whereas Aβ alone induces the polymerization. Further, Aβ decreases gelsolin levels, and the latter is involved in Aβ removal. Our data indicates that Aβ-laminin and Aβ-gelsolin complexes are less neurotoxic and also less potent than fibrillar Aβ at inducing isAPP retention. These results validate the potential of these proteins as therapeutic strategies that prevent the Aβ-induced effects. In hence, given that Aβ decreases the levels of proteins involved in its own clearance, this may contribute to the mechanisms underlying AD pathology.

## Introduction

The deposition and accumulation of aggregates of fibrillar Abeta (Aβ) peptides are considered one of the key processes underlying neuronal loss in Alzheimer’s disease (AD) pathology. The resulting senile plaques are mainly composed of Aβ (Glenner and Wong 1984) but also include inflammatory molecules, proteoglycans, metal ions, antioxidant proteins and proteases, as well as clearance-related compounds (Atwood et al. 2002). However, Aβ exists in normal brains and cerebrospinal fluid (CSF) suggesting that it may have a relevant physiological function. Indeed, some studies have reported that Aβ monomers may be neuroprotective and may support neuronal survival (Soucek et al. 2003; Brody et al. 2008; Giuffrida et al. 2009), which is also in agreement with the observations that many aged individuals, despite the presence of senile plaques, show little or no cognitive decline. Further, some authors propose that rather than be the trigger of disease pathogenesis, Aβ may function as a protective adaptation to disease (Lee et al. 2004) and that its accumulation and deposition into senile plaque may be a protective event.

Several investigators have reported that Aβ neurotoxicity is related to the degree of fibrillization, the β-sheet structure, and the size of the peptides (Pike et al. 1993; Lorenzo and Yankner 1994; Dahlgren et al. 2002). The molecular mechanisms responsible for the passage of normal soluble Aβ forms to fibrils are not well understood. Nonetheless, it is known that Aβ binds several proteins that can modulate its aggregation or its fibrillar state thus attenuating its neurotoxicity. Among these are laminin (Bronfman et al. 1996; Drouet et al. 1999; Monji et al. 1999; Morgan et al. 2002) and gelsolin (Chauhan et al. 1999, 2008; Ray et al. 2000; Qiao et al. 2005), which have been shown to bind and form complexes with the Aβ peptide. Laminin is a major basement membrane protein shown to inhibit Aβ fibril formation and to disaggregate preformed Aβ fibrils (Monji et al. 1999; Morgan and Inestrosa 2001; Morgan et al. 2002). Gelsolin is found both as an intracellular protein (Tanaka and Sobue 1994; Ji et al. 2008), which is able to modulate actin assembly and disassembly (Janmey et al. 1985; Howard et al. 1990), and as a secreted plasma and CSF protein (Kwiatkowski et al. 1988; Paunio 1994; Kulakowska et al. 2008). Like laminin, it was reported to prevent Aβ fibrillogenesis and to defibrillize preformed fibrils (Ray et al. 2000; Chauhan et al. 2008; Carro 2010).

Aβ is an intracellular product of the proteolytic processing of the Alzheimer’s amyloid precursor protein (APP) (da Cruz e Silva and da Cruz e Silva 2003), generated by sequential cleavage by α-secretase (mainly BACE1) (Gandy et al. 1994; Vassar et al. 1999; Yan et al. 2001) and the γ-secretase complex (reviewed by Kaether et al. 2006). The physiological function of Aβ is unclear, but it is known to trigger many cellular responses. Among the most devastating is its ability to trigger several apoptotic and neurotoxic mechanisms and hence to promote cellular death (Pereira et al. 2004; Kaminsky et al. 2010). Other reports have also associated Αβ to altered APP metabolism (Davis-Salinas et al. 1995; Schmitt et al. 1997; Moreno-Flores et al. 1998; Carlson et al. 2000; Henriques et al. 2009a, 2010). Surprisingly, Aβ was to modulate APP metabolism, toward the nonamyloidogenic α-secretase pathway. In our previous work, Aβ was able to prevent secretion of the neurotrophic and neuroprotective secreted APP α fragment (sAPPα) (Henriques et al. 2009a) and to decrease AICD production, consequently decreasing APP expression levels (Henriques et al. 2009b), thus keeping “in check” its own production. This Aβ-induced effect was also associated with abnormalities in the cytoskeletal network (Henriques et al. 2009a, 2010).

It seems reasonable to deduce that in AD, some of these control events are behaving anomalously, forcing the need to identify potential therapeutic targets. In the work presented here, we monitored molecular relevant events: Aβ aggregation and intracellular secreted APP (isAPP) production in the presence of gelsolin and laminin. Our data demonstrated that inhibition of Aβ fibril formation by laminin or gelsolin was able to partially revert Aβ-induced effects on APP metabolism, decreasing is APP and reducing Aβ-induced actin polymerization, thus suggesting a potential therapeutic avenue for these Aβ complexing proteins.

## Materials and Methods

Laminin (from basement membrane of Engelbreth-Holm-Swarm mouse carcinoma) and gelsolin (from bovine plasma) were purchased from Sigma. Gelsolin was prepared as a 10 μM stock dissolved in 25 mM Tris-HCl, pH 7.5. Aβ_25–35_ (Sigma), Aβ_1–40_ and Aβ_1–42_ peptides (American Peptide Company), and Aβ_25–35_ with a scrambled sequence (Aβ_scrb,_ from NeoMPS) were dissolved in sterile distilled water to obtain a 1 mM stock solution. Thioflavine T (Sigma) was prepared as a 3 mM stock in distilled water.

The monoclonal antibody 22C11 directed against the APP N-terminus (Boehringer) was used in this study to detect APP and total sAPP. Holo-APP was detected using the APP C-terminal antibody (rabbit polyclonal anti-β-APP C-terminus, Zymed). Monoclonal anti-gelsolin antibody (Clone GS-2C4, Sigma) was used to detect the approximately 85 KDa gelsolin band. The monoclonal anti-β-tubulin antibody (Amersham Pharmacia) was used as a control. Immunoreactive bands were detected by enhanced chemiluminescence (ECL or ECL plus for sAPP detection, Amersham Pharmacia).

### Aβ Aggregation

Aβ aggregation was achieved by incubating the different peptides for 48 h at 37 °C in phosphate-buffered saline (PBS, pH 7.4). Prior to adding to the cells, Aβ was incubated with laminin or gelsolin. For coincubation assays, 100 μM Aβ_25–35_ was incubated in the presence of either laminin at 0.5 μM (Aβ/laminin molar ratio of 200:1) or gelsolin at 2.2 μM (Aβ/gelsolin molar ratio of 45:1) in PBS for 48 h at 37 °C, concentrations around those previously reported studies (Monji et al. 1999; Ray et al. 2000).

Fibrillar peptide and peptide complexes were then diluted in medium, before adding to the cells in culture (see “Aβ treatment section).

### Thioflavine T (Th-T) Fluorometric Assay

Aβ_25–35_ fibril formation in the presence or absence of the proteins mentioned above was monitored by a Th-T fluorometric assay as described by LeVine (1993), with a few modifications. Th-T fluorescence was measured at 0 and 48 h of incubation at 37 °C, using a Jasco FP-777 spectrofluorometer. Th-T measurements used 20 μl of incubated sample mixed with 980 μl and 15 μM Th-T in PBS. Samples were monitored at λ_Ex_ = 450 nm and 5 nM bandpass, and λ_Em_ = 482 nm and 10 nM bandpass. The background fluorescence of Th-T and buffer alone were subtracted from the fluorescence value obtained for each sample.

### Cell Culture

Rat cortical neuronal cell cultures were prepared from embryonic day 18 Wistar rat fetuses as previously described (Henriques et al. 2007). Briefly, after dissociation with trypsin (1.0 mg/ml; 10 min; 37 °C), the cells were plated on poly-d-lysine-coated dishes at a density of 1.0 × 10^5^ cells/cm^2^ in B27-supplemented Neurobasal medium (Gibco), a serum-free medium combination, supplemented with glutamine (0.5 mM) and gentamicin (60 μg/ml). Cultures were maintained in an atmosphere of 5 % CO_2_ at 37 °C during 9 days before being subjected to the various experimental conditions described.

HeLa cells (ATTC CRM-CCL-2) were grown in Dulbecco’s Modified Eagle Medium (DMEM), supplemented with 10 % fetal bovine serum, 1 % antibiotic-antimycotic, and NaHCO_3_ at 3.7 g/l.

### Aβ Treatment

**Fig. 1:**
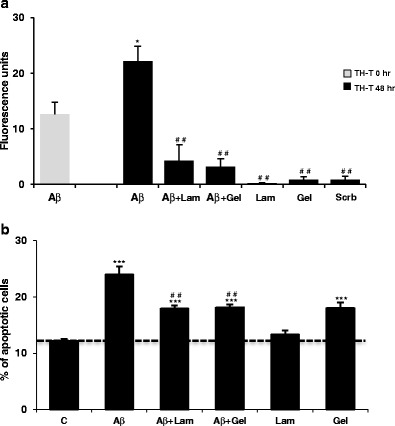
Effect of laminin and gelsolin on Aβ aggregation and toxicity. **a** Aβ_25–35_ was incubated at 37 °C for 48 h, either alone or in the presence of 0.5 μM laminin (Lam) or 2.2 μM gelsolin (Gel), and the fluorescence intensity of Th-T was measured at λex = 450 nm and λem = 482 nm. Results are expressed as mean ± SEM of at least three independent experiments. ^##^ *P* < 0.01, significantly different from Aβ 48 h; ^*^ *P* < 0.05 significantly different between 0 and 48 h Aβ treatment, Tukey post hoc test. **b** Aβ_25–35_ apoptotic induction in the presence of laminin and gelsolin was determined using Hoechst stain. Cortical neurons were visualized, and the percentage of apoptotic nuclei for each condition was determined relative to control. *C* control, *Lam* laminin, *Gel* gelsolin. ^##^ *P* < 0.01, significantly different from Aβ; ^***^ *P* < 0.001, significantly different from control; Tukey post hoc test

Exposure of cells to Aβ was preceded by an aggregation step and the effect of different Aβ peptides and concentrations was evaluated (Fig. 1). Subsequent experiments were carried out by preincubating Aβ_25–35_ peptide alone or in the presence of the different proteins (laminin and gelsolin) during 48 h at 37 °C; 100 μl of each preparation were then diluted in 900 μl of the complete corresponding medium and added to the cells for a 24 h period. Under these conditions, Aβ was used at a final concentration of 10 μM, laminin at 50 nM, and gelsolin at 220 nM.

To evaluate gelsolin levels, HeLa cells were treated with 10 μM of aggregated Aβ_25–35_ and Aβ_1–42_ for 24 h in a DMEM serum-free combination.

### Monitoring Apoptosis

In order to morphologically evaluate apoptosis, cells were plated at a density of 0.8 × 10^5^ cells/cm^2^ on poly-d-lysine-coated coverslips. After the appropriate treatments, cells were fixed with 4 % paraformaldehyde diluted in media (1:1) for 2 min and then incubated for 15 min in undiluted paraformaldehyde. Following three washes with PBS, cells were incubated with Hoechst dye 33258 (5 mg/ml stock, Sigma) at 5 μg/ml in PBS for 3 min to allow for nuclear staining. For quantitative analysis of apoptosis, cell nuclei were visualized using a 4′,6-diamidino-2-phenylindole (DAPI) filter in an Olympus IX-81 inverted epifluorescence microscope. To evaluate the percentage of apoptotic cells, ten independent fields were analyzed (approximately 400 cells) per experimental condition. Under control conditions, primary neuronal cultures exhibited approximately 10 % of apoptotic cells.

### F-actin Labeling

Subsequent to the different experimental conditions, HeLa cells were fixed with paraformaldehyde and permeabilized with a cold solution of acetone (−20 °C) for 3 min. Cells were then washed with PBS and subsequently incubated with PBS containing 1 % bovine serum albumin (BSA) for 1 h. This blocking solution was removed, and phallotoxin staining solution (labels F-actin) was added to the cells (1.5 U/100 lL in PBS containing 1 % BSA) for 20 min at 25 °C. After PBS washing, the coverslips were mounted on microscope glass slides using antifading reagents containing DAPI for nucleic acids (Vectashield, Vector Laboratories, Burlingame, CA). To evaluate the effects on F-actin polymerization, 15 independent fields (approximately 60 cells) were analyzed per experimental condition of independent experiments. Epifluorescence images were acquired with an Olympus IX-81 motorized inverted epifluorescence microscope equipped with the appropriate filter combinations and a 100× objective (Plan-Neofluar, 100×/1.35 oil objective). Photographs were acquired with a Digital CCD monochrome camera F-View II (Soft Imaging System) and processed with the AnalySIS software (Soft Imaging System) (Vieira et al. 2009).

### Sample Collection and Protein Analysis

Following appropriate treatments, conditioned media and cells were collected into boiling 1 % sodium dodecyl sulfate (SDS). The cell lysates were homogenized as previously described (Amador et al. 2004). Protein determination of the lysates was carried out with the BCA kit (Pierce). Samples were normalized for protein content; 50 μg of each lysate was loaded and separated on a 7.5 or 5–20 % gradient (for gelsolin experiments) SDS-PAGE and then electrophoretically transferred onto a nitrocellulose membrane. Immunoblotting was carried out with the C-terminal antibody for holo-APP (hAPP) detection or the 22C11 antibody, which detects hAPP and isAPP (intracellular sAPP). Anti-gelsolin and anti-β-tubulin antibodies for detecting gelsolin and tubulin, respectively, were also used. Medium-secreted/extracellular sAPP (esAPP) was detected by subjecting the conditioned medium to Western blot analysis and using the 22C11 antibody. Detection was achieved using ECL (for hAPP, gelsolin, and tubulin) or ECL plus (for sAPP) detection systems.

### Quantification and Statistical Analysis

Quantity One densitometry software (Bio-Rad) was used to scan and quantify immunoblot band intensity. Data are expressed as mean ± SEM of at least three independent experiments. Statistical analysis was carried out using one-way analysis of variance (ANOVA). When the *F* values were significant, the Tukey test was applied to compare all pairs of groups. For gelsolin experiments, one sample *t* test analysis was performed. Statistical differences have been determined at *P* < 0.05.

## Results and Discussion

### Laminin and Gelsolin Effects on Aβ_25–35_ Aggregation and Neurotoxicity

Aβ toxicity is closely associated with its fibril state; hence, proteins which can modulate the latter may potentially prevent Aβ amyloidogenic responses. Previous reports showed that laminin (Drouet et al. 1999; Monji et al. 1999; Morgan et al. 2002) and gelsolin (Ray et al. 2000; Qiao et al. 2005) inhibit fibril formation or depolymerize preformed fibrils of both Aβ_1–40_ and Aβ_1–42_ peptides. The effects of both these proteins on Aβ_25–35_ aggregation and subsequent effects on APP metabolism were evaluated in this study.

To quantify the extent to which Aβ_25–35_ aggregates, the Th-T fluorescence assay was used. Although Aβ_25–35_ spontaneously aggregates in solution, an additional aggregation period of 48 h at 37 °C resulted in an increase of the relative Th-T fluorescence intensity (Fig. 1a) from 12 to 22 fluorescence units. The procedure was repeated, but in the presence of laminin and gelsolin in a molar ratio of 200:1 for Aβ and laminin and 45:1 for Aβ and gelsolin, concentrations chosen were near those previously employed (Monji et al. 1999; Ray et al. 2000). By comparing with the incubation of Aβ alone, a significant decrease in the fluorescence of Th-T was obtained when Aβ_25–35_ was coincubated with laminin or gelsolin for 48 h (from 22 fluorescence units to 4 and 3, respectively). Hence, as expected in the presence of these two proteins, Aβ_25–35_ fibril formation was dramatically inhibited (Fig. 1a). Data also indicate that both laminin and gelsolin bind Aβ within the Aβ_25–35_ region.

Laminin and gelsolin inhibition of fibril formation have been associated with a decrease in Aβ-induced neurotoxicity (Morgan et al. 2002; Qiao et al. 2005; Carro 2010). Like the full-length Aβ peptides, Aβ_25–35_ was previously reported to induce apoptosis, thus neurotoxicity of Aβ and Aβ-complexes was evaluated, by quantitative analysis of cell nuclei following Hoechst staining. The percentage of picnotic/apoptotic cell nuclei was estimated, for primary cortical cultures when exposed to 10 μM of Aβ_25–35_, both in the absence and presence of laminin (50 nM) or gelsolin (220 nM) for 24 h (Fig. 1b). The levels of apoptosis induced by Aβ peptide alone were low (∼15 % above control levels). Of note, the levels could be decreased to ∼5 % above control levels, if Aβ was preincubated with either laminin or gelsolin. Thus, indicating that laminin and gelsolin can significantly attenuate the toxic effects of Aβ_25–35_ as deduced from the decrease in Aβ-induced apoptosis.

### Effect of Aβ-Laminin and Aβ-Gelsolin Complexes on APP Metabolism

**Fig. 2:**
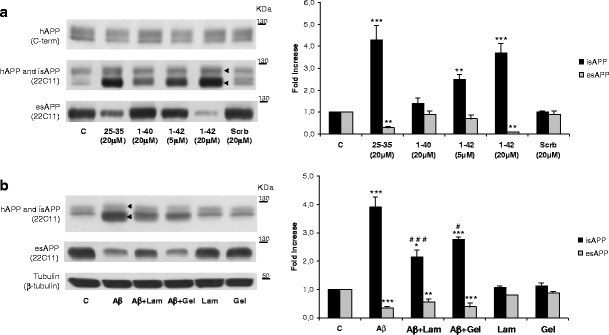
Aβ-laminin and Aβ-gelsolin complexes revert Aβ-induced anomalies in neuronal APP processing. **a** Aβ_25–35_, Aβ_1–40_, and Aβ_1–42_ were aggregated for 48 h at 37 °C. Following aggregation, peptides were added to primary neuronal cultures at different concentrations for 24 h. Scramble 25–35 peptide was used as a control (*Scrb*). **b** Primary cortical cultures were incubated for 24 h with 10 μM Aβ in the absence or presence of laminin (*Lam*) or gelsolin (*Gel*). Intracellular holo-APP (hAPP) protein was detected by subjecting the cellular lysates to Western blot analysis with the C-terminal antibody. The levels of intracellular hAPP and sAPP (isAPP) and extracellular (medium secreted) sAPP (esAPP) were detected using the N-terminal antibody 22C11. *Left-pointing pointer*, isAPP retention, corresponds to protein bands immunoreactively positive for 22C11 but negative for C-terminal antibody. Quantitative results are expressed as mean ± SEM of three independent experiments. Fold increase in isAPP and esAPP; ^#^ *P* < 0.01 and ^###^ *P* < 0.01, significantly different from Aβ; ^*^ *P* < 0.05, ^**^ *P* < 0.01, and ^***^ *P* < 0.001 significantly different from control; Tukey post hoc test

As discussed above, the Aβ binding proteins (laminin and gelsolin) prevent fibrillogenesis rendering Aβ less neurotoxic. Consequently, we took advantage of this effect, to evaluate whether Aβ-induced abnormal APP processing could also fluctuate in response to the aggregation state of the former. Hence, the Aβ protein complexes were added to cultured cells, and APP metabolism was evaluated. When primary cortical cultures were treated with 10 μM of aggregated Aβ_25–35_ for 24 h, an approximately fourfold increase in intracellular sAPP (isAPP) levels was observed (Fig. 2a) as detected by using the N-terminal APP 22C11 antibody. This antibody detects both sAPP and hAPP, but since hAPP levels did not change in response to Aβ (C-terminal detection, Fig. 2a), the observed increase was due to isAPP accumulation. This was previously shown by our group for non previously aggregated Aβ. Regarding the naturally produced Aβ peptides, at 20 μM concentration, Aβ_1–40_ peptide was much less effective at inducing an increase in isAPP than Aβ_25–35_ and Aβ_1–42_ peptides. Consistent with the previous observations, medium-secreted extracellular sAPP (esAPP) decreased upon exposure to Aβ, once again with the exception of Aβ_1–40_ (20 μM). This data is in agreement with a decrease propensity to aggregate and a less toxic effect of the Aβ_1–40_ peptide when compared with the Aβ_1–42_. This peptide species and concentration-dependent effect were mirrored in the increase in isAPP retention (Fig. 2a). The scramble Aβ_25–35_ peptide was used as a control and, in fact, confirmed the specificity of the Aβ response.

However, in the presence of Aβ-laminin and Aβ-gelsolin complexes, the levels of isAPP decreased to 2.0-fold and 2.5-fold, respectively, when compared with Aβ treatment alone (Fig. 2b). Thus, by decreasing Aβ fibrillogenesis with both laminin and gelsolin, one promotes less toxic Aβ species, resulting in a decrease in Aβ-induced isAPP accumulation. Laminin and gelsolin alone showed no effect on APP intracellular levels (Fig. 2b).

With respect to medium-secreted APP (esAPP), we observed that aggregated Aβ_25–35_ resulted in decreased total esAPP levels. For the proteins tested, it would be expected that diminishing isAPP would result in increased esAPP secretion. This was evident for laminin, which induced a slight increase on esAPP (from 0.3 to around 0.5), thus attenuating the effect of Aβ alone. Laminin was previously shown to decrease sAPP secretion and to affect intracellular APP biogenesis and catabolism (Monning et al. 1995; Bronfman et al. 1996). Therefore, we would not expect a complete reversion of the sAPP secretion. The reversion on sAPP secretion was not significant with Aβ-gelsolin complexes. This may reflect different modes of action for laminin and gelsolin or simply that the Aβ-protein ratios used were not sufficient to revert the Aβ-induced effects. Overall, under the conditions tested, the Aβ effects on APP processing were reversed or attenuated, with respect to isAPP accumulation for gelsolin and laminin, and the latter also partially reverted the decrease in esAPP, which is a typical response following Aβ exposure.

### F-actin Polymerization upon Aβ-laminin and Aβ-gelsolin Complexes

**Fig. 3:**
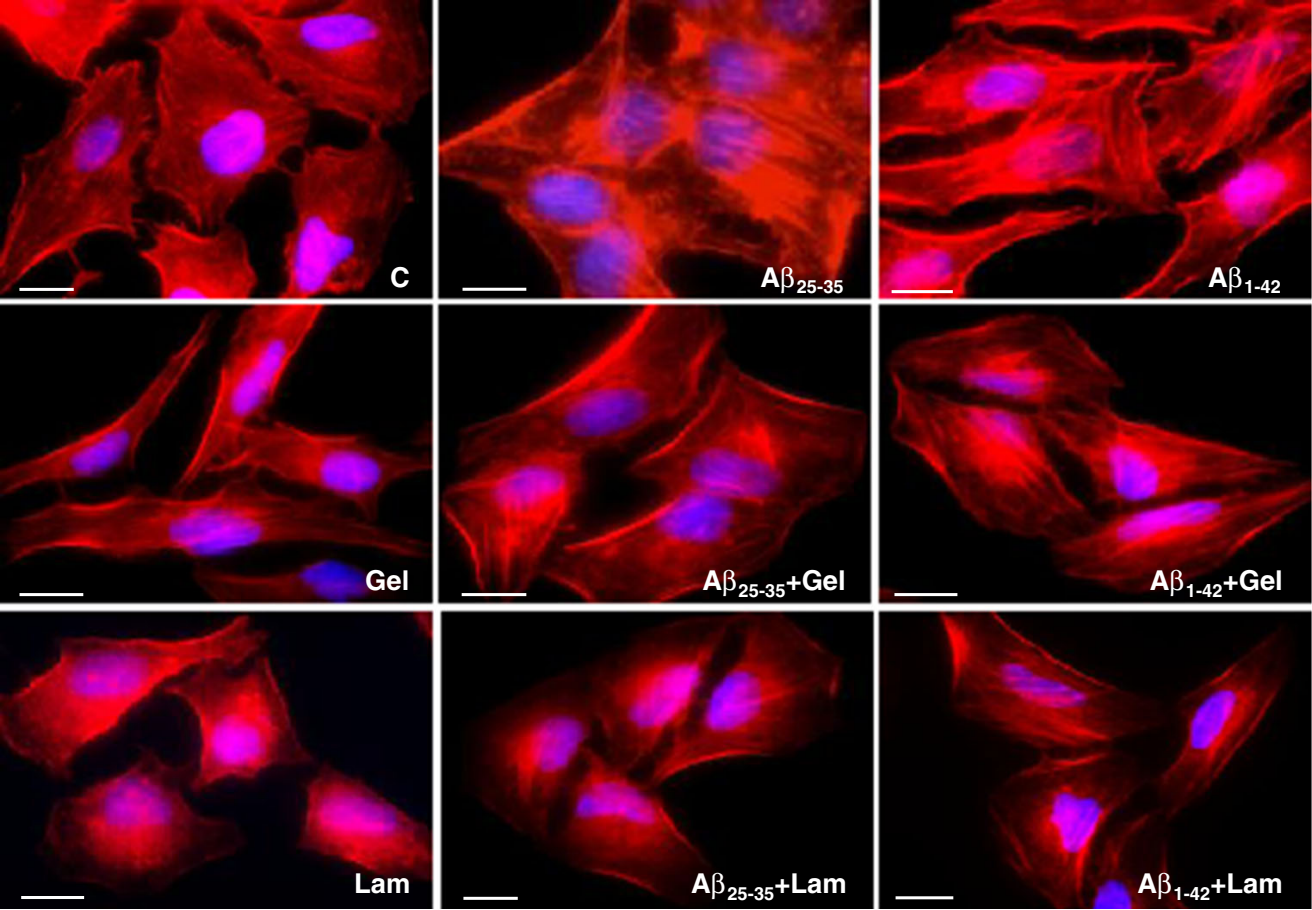
Aβ-laminin and Aβ-gelsolin complexes on F-actin polymerization. Upon incubation for 24 h with either Aβ_25–35_ or Aβ_1–42_, HeLa cells were stained with Alexa Fluor 568 conjugated phallotoxin solution (labeled filamentous actin, F-actin). Images were acquired using an Olympus IX-81 microscope. *C* control, *Lam* laminin, *Gel* gelsolin. *Bar*, 10 μm

Aβ effects on isAPP retention were previously correlated with abnormalities in the cytoskeletal network, in particular in both actin and tubulin networks, since both actin depolymerization and microtubule stabilization agents could partially revert the Aβ-induced isAPP retention (Henriques et al. 2010). In the present study, we evaluate the F-actin polymerization in the presence and absence of Aβ-laminin and Aβ-gelsolin complexes (Fig. 3). Under control conditions, we could observe actin fibers, but these are much shorter and thinner when compared with conditions in the presence of Aβ peptide. In fact, Aβ exposure led to an increase in F-actin polymerization and in the thickness of the actin stress fibers formed. Monomeric G-actin is in dynamic equilibrium with polymerized F-actin. Gelsolin is an actin-capping protein, which may regulate actin polymerization by binding at the plus ends, therefore preventing elongation. However, rapid signal cascades can induce actin polymerization and reverse the entire process. F-actin filaments tend to be slightly decreased below the plasma membrane, in particular for laminin treatment comparative to control, which may be correlated with a slight decrease in sAPP secretion for those proteins (Fig. 2b). The data suggest that a basal level of F-actin polymerization is needed for sAPP secretion, otherwise, an enormous increase in sAPP secretion should be expected under actin depolymerization conditions. In agreement, previous data from our group showed that incubation with an actin-depolymerizing agent only partially reverse the inhibition of sAPP secretion induced by Aβ (Henriques et al. 2010).

Aβ-laminin and Aβ-gelsolin complexes result in decreased F-actin polymerized filaments (Fig. 3) when compared with Aβ conditions. The cytoskeletal alterations induced by Aβ could be partially reverted by both Aβ-laminin and Aβ-gelsolin complexes. Intracellular sAPP retention decreased in both cases, but contrary to what was expected, only Aβ-laminin complexes result in a slight increase of esAPP (Fig. 2b). This may correlate with the individual effects of the proteins alone in F-actin  remodelling/dynamics, which can render in decreased esAPP. Presently, it is difficult to interpret the significance of the decrease in isAPP levels toward control conditions, induced by these Aβ-protein complexes, because the metabolism and clearance mechanisms of the compounds tested are not completely understood. Nonetheless, we postulate that the laminin- or gelsolin-Aβ complexes formed may inhibit Aβ-mediated signaling and/or be intracellularly targeted for degradation, therefore preventing Aβ-induced responses. Of note, the sAPP fragment resulting from the nonamyloidogenic pathway was reported to have neurotrophic and neuroprotective properties (Mattson et al. 1993; Meziane et al. 1998); hence, isAPP accumulation may contribute to neuronal degeneration. Therefore, modulation of sAPP intracellular accumulation and secretion may thus be a protective event.

### Aβ Effects on Gelsolin Levels

**Fig. 4:**
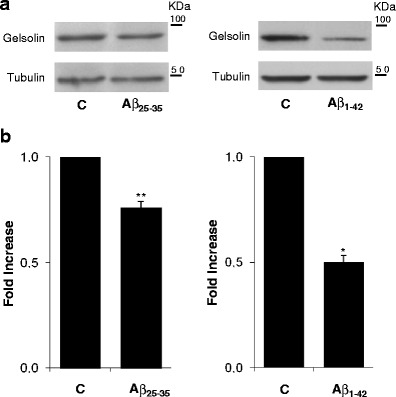
Gelsolin levels in response to Aβ. HeLa cells were incubated for 24 h with Aβ_25–35_ and Aβ_1–42_, both at 10 μM and aggregated. **a** Intracellular gelsolin levels were monitored by Western blot analysis using anti-gelsolin antibody. Tubulin was used as an internal control. **b** Quantitative results are expressed as a fold increase relative to the basal levels. *C* control. ^*^ *P* < 0.05 and ^**^ *P* < 0.01, significantly different from control; One sample *t* test

Due to their depolymerization properties and their capacity to bind Aβ, both laminin and gelsolin could potentially alter the Aβ periphery; brain load, by promoting the reduction of this toxic peptide in the brain. In particular, peripheral administration of plasma gelsolin resulted in a reduction of Aβ_1–40_/Aβ_1–42_ and Aβ load in the brains of APP/PS transgenic mice that exhibited elevated intraneuronal Aβ_1–42_ levels (Matsuoka et al. 2003). Moreover, in similar transgenic mouse models of AD, peripheral delivery of plasmid DNA encoding plasma gelsolin, also led to a reduction of Aβ levels in the brain (Hirko et al. 2007). The reduction in Aβ pathology appeared to be related to an increase in activated and reactive microglia and to soluble oligomeric forms. Also, it has been previously reported that plasma gelsolin levels were decreased in AD patients (Guntert et al. 2010). Herein, we show that both Aβ_25–35_ and Aβ_1–42_ can decrease the levels of this Aβ-disaggregating protein (Fig. 4). Hence, the Aβ clearance mechanisms prompted by gelsolin may be dysregulated by the Aβ peptide itself, which can contribute to its own deposition and accumulation in AD brains. Hence, Aβ-disaggregating proteins can be relevant in the development of future therapeutic strategies designed to act at the level of Aβ aggregation and accumulation.

**Fig. 5:**
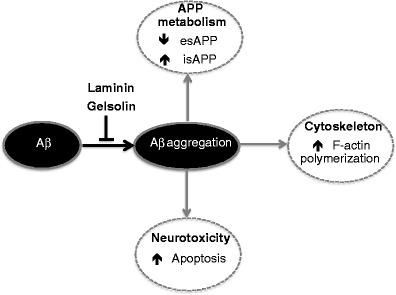
Schematic representation of the Aβ-disaggregating proteins’ mode of action. Aggregated Aβ induces neurotoxicity, altered APP processing and cytoskeletal rearrangements, which can be reversed by laminin and gelsolin. These proteins are able to inhibit Aβ fibril formation and aggregation or to defibrillize its preformed fibrils, thus preventing Aβ-mediated effects

Overall, this study showed that both laminin and gelsolin were able to revert Aβ effects not only at the neurotoxic level but also at the metabolic level. Besides reverting isAPP levels, laminin could also increase esAPP secretion, which may counteract the Aβ-induced neurotoxic effects. This response could be related to a decrease in F-actin polymerization, which could also be induced by Aβ-disaggregating protein complexes. At the molecular level, by preventing Aβ aggregation, these proteins may exert their effect at several distinct stages (Fig. 5), namely, by interfering with Aβ-induced cytoskeletal alterations and isAPP retention. Of note, gelsolin was reported to be involved in Aβ clearance in the brain. Since we show that the latter peptide was able to decrease gelsolin levels, this may represent a pathway by which Aβ may contribute to its own brain accumulation. Although the molecular events underlying the mode of action of both proteins are unknown, our data suggest that both Aβ-disaggregating proteins may be of therapeutic value for AD pathology.
